# Supplementary material for: Reliability of task-based fMRI in the dorsal horn of the human spinal cord
Source: Imaging Neurosci (Camb). 2024 Aug 22;2:imag-2-00273. doi: 10.1162/imag_a_00273 (PMC12290578; doi:10.1162/imag_a_00273)
Supplement: Supplementary Material [file imag_a_00273-supp.pdf]

Supplementary Material

Reliability of task-based fMRI in the dorsal horn of the human spinal cord

Alice Dabbagh, Ulrike Horn, Merve Kaptan, Toralf Mildner, Roland Müller, Jöran Lepsien, Nikolaus Weiskopf, Jonathan C.W. Brooks, Jürgen Finsterbusch, Falk Eippert

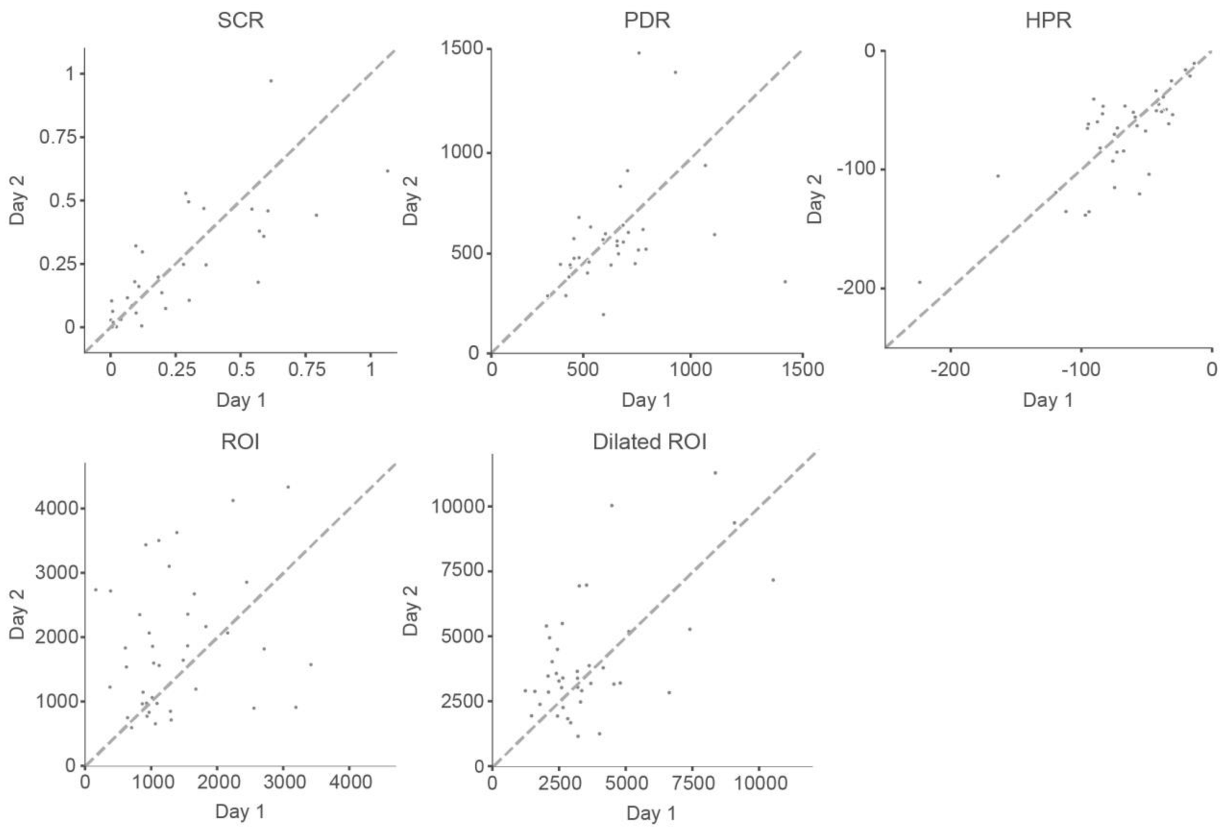

Supplementary Figure 1. Individual values underlying ICC calculation. Participant-wise data of Day 1 and Day 2 peak values for skin conductance responses (SCR), pupil dilation responses (PDR) and heart period responses (HPR), as well as average top 10% β values of the left dorsal horn (ROI) and the dilated left dorsal quadrant (Dilated ROI) in spinal cord segment C6.

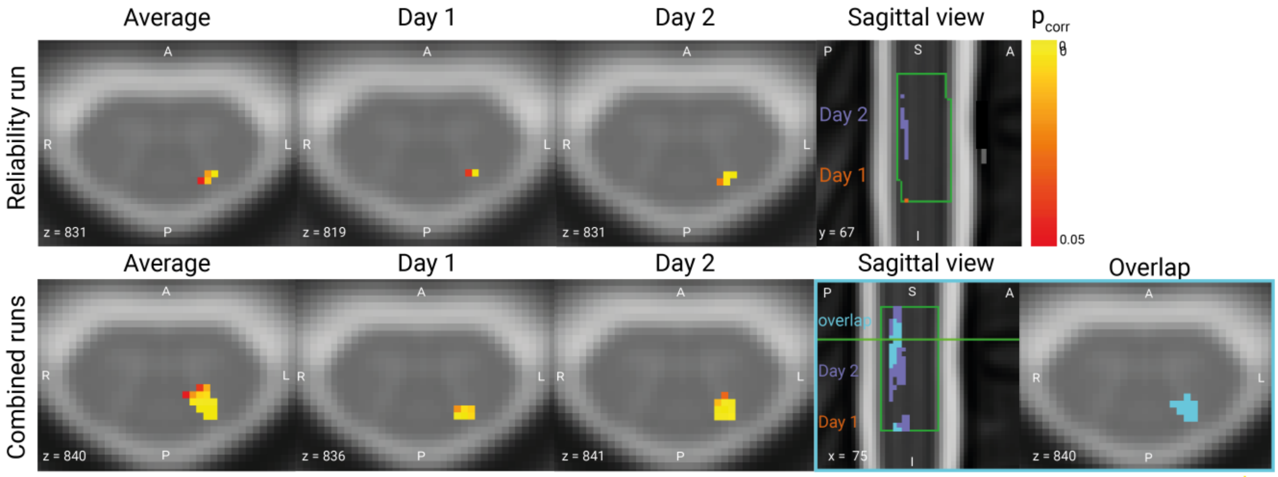

Supplementary Figure 2. Group-level fMRI results. Results are shown for the Reliability Run (i.e., one run per day; top row; same image as in main manuscript used for comparison purposes here) and Combined Runs (i.e. average of four runs per day; bottom row). Only voxels surviving a threshold of $p < 0.05$ (corrected for multiple comparisons via a permutation test in a mask of the left dorsal horn in spinal cord segment C6) are displayed on top of a T2*-weighted spinal cord template (PAM50) in axial view, and on top of a T2-weighted spinal cord template (PAM50) in the sagittal view. In contrast to the Reliability Run, the Combined Runs demonstrated substantial overlap across both days. Spinal cord segment C6 is outlined in green in the sagittal images.

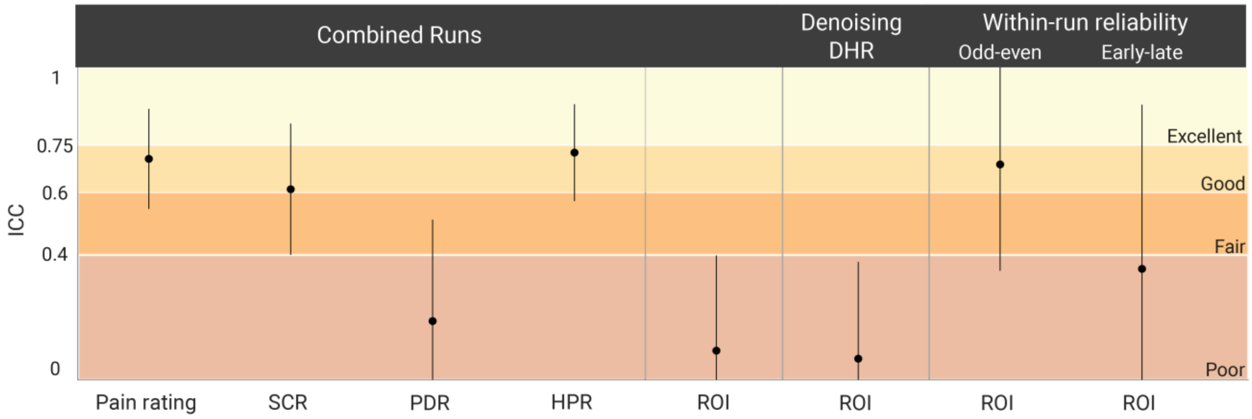

Supplementary Figure 3. Test-retest reliability across both days for subjective ratings, peripheral physiological data and BOLD response amplitudes. Reliability is indicated via ICCs (plotted as dots with 95% CI represented as a line). *Combined Runs*: ICCs are reported for (from left to right) verbal ratings, SCR, PDR, HPR and top 10% β-estimate in the left dorsal horn of C6 (ROI). *Denoising DHR*: ICC of the top 10% β-estimate in the left dorsal horn of C6 (ROI) of only the Reliability Run, obtained from a GLM with an additional regressor of right dorsal horn activity. *Within-run reliability*: ICCs obtained from comparing either odd and even trials numbers (odd-even), or the first and second half of a run (early-late). Colors indicate ICC interpretation according to Cicchetti (1994): dark red: ICC < 0.4, poor; medium red: ICC 0.4 - 0.59, fair; orange: ICC 0.6 - 0.74, good; yellow: ICC 0.75 - 1.0, excellent.

Supplementary Table 1

Percent suprathreshold voxels in the four cord quadrants of each spinal segment.

Spinal cord segment	Total number of active voxels ($p < 0.001$)	DL	DR	VL	VR
Average					
C5	1140	50.1%	34.5%	5.6%	9.8%
C6	674	61.3%	38.4%	0.3%	0%
C7	604	14.6%	26.7%	13.4%	45.4%
C8	62	22.6%	56.5%	8.1%	12.9%
Day 1					
C5	438	79.0%	14.4%	4.6%	2.1%
C6	65	83.1%	10.8%	6.2%	0%
C7	59	37.3%	25.4%	5.1%	32.2%
C8	3	0%	100%	0%	0%
Day 2					
C5	302	28.1%	49.7%	4.0%	18.2%
C6	332	66.3%	27.7%	5.1%	0.9%
C7	235	9.8%	11.9%	48.9%	29.4%
C8	17	0%	82.4%	0%	17.6%

Notes. Results are based on the group-level results of each day's Reliability Run. ROI names refer to spinal cord quadrants in the respective segment. *Abbreviations*: dorsal left (DL), dorsal right (DR), ventral left (VL), ventral right (VR). This analysis was carried out using the masks of the four cord quadrants separately for each spinal segment.

Supplementary Table 2

Intraclass correlation coefficient and 95% confidence interval for subjective ratings, peripheral physiological data and BOLD response amplitudes of post-hoc analyses.

Change in analysis pipeline	Measures			ICC (95% CI)
Combined runs average	Ratings			0.71 (0.51–0.83)
	SCR			0.61 (0.36–0.78)
	PDR			0.19 (-0.16–0.81)
	HPR			0.73 (0.54–0.85)
	DH left	β	peak	0.20 (-0.12–0.48)
			Top 10%	0.09 (-0.22–0.39)
			avg	-0.08 (-0.38–0.23)
		z-score	peak	0.07 (-0.24–0.37)
			Top 10%	-0.01 (-0.31–0.30)
			avg	0.03 (-0.28–0.34)
DHR regressor	DH left	β	peak	0.11 (-0.21–0.40)
			Top 10%	0.08 (-0.25–0.37)
			avg	-0.07 (-0.37–0.24)
		z-score	peak	0.11 (-0.2–0.41)
			Top 10%	0.06 (-0.25–0.36)
			avg	0.025 (-0.29–0.33)
Within-run reliability				
Odd-even	SCR			0.81 (0.67–0.90)
	PDR			0.82 (0.66–0.90)
	HPR			0.86 (0.76–0.93)
	DH left	β	peak	0.63 (0.41–0.79)
			Top 10%	0.69 (0.49–0.83)
			avg	0.52 (0.27–0.71)
Early-late	SCR			0.56 (0.31–0.74)
	PDR			0.61 (0.35–0.78)
	HPR			0.79 (0.64–0.89)
	DH left	β	peak	0.31 (0.02–0.56)
			Top 10%	0.36 (0.07–0.59)
			avg	-0.003 (-0.31–0.31)

Abbreviations: skin conductance response (SCR), pupil dilation response (PDR), heart period response (HPR), dorsal horn (DH), right dorsal horn (DHR) in spinal cord segment C6.

Supplementary Table 3

Correlations between response measures.

Peripheral / subjective measure	BOLD parameter estimate (top 10%)	Pearson's r	p-value
			One-tailed
Ratings	β	-0.18	0.855
	z-score	-0.30	0.965
SCR	β	0.34	0.017 *
	z-score	0.36	0.014 *
HPR	β	-0.28	0.038 *
	z-score	-0.28	0.039 *
PDR	β	-0.11	0.722
	z-score	-0.05	0.611

Notes. Results are based on individual BOLD responses and peripheral physiological responses and subjective ratings of the reliability run. We correlated responses averaged across days for each measure. BOLD responses were quantified as the top 10 % of β estimates and z-scores extracted from the left dorsal horn in spinal cord segment C6. *Abbreviations*: skin conductance response (SCR), pupil dilation response (PDR), heart period response (HPR). For further information see section 2.8.4. * $p < 0.05$

Supplementary Table 4

Correlations between BOLD parameter estimates and indicators of data quality.

Data quality estimate	BOLD parameter estimate (top 10%)	Pearson's r	p-value
			One-tailed
Motion	β	0.16	0.159
	z-score	0.19	0.118
Normalization quality	β	-0.25	0.939
	z-score	-0.14	0.8
Angulation relative to B0	β	0.41	0.004 **
	z-score	0.40	0.005 **

Notes. Results are based on individual BOLD responses and data quality indicators of the reliability run. We correlated absolute differences across days for each measure. BOLD responses were quantified as the top 10 % of β estimates and z-scores extracted from the left dorsal horn in spinal cord segment C6. Motion was quantified as root mean square intensity differences of each volume to reference volume. Normalization quality was quantified as the Dice coefficient between the segmentation of the normalized mean functional image and the PAM50 cord mask across the same z-range. The angulation relative to B0 describes the angle between the scanner's z-axis (aligned with B0) and the z direction in the slice-stack. For further information see section 2.8.5. * $p < 0.05$, ** $p < 0.01$

Deviations from preregistration

In the preregistration, we stated that in addition to ICC(3,1) we would report the Pearson correlation coefficient and ICC(2,1) as indicators of reliability. However, for the sake of brevity we ultimately decided to report only ICC(3,1), as all indicators were found to be highly similar.

In the preregistration, we stated that we aimed to calculate voxel-wise ICC maps. However, given that we observed almost no overlap of activation across days in the analysis reported here (thus making a voxel-wise assessment pointless), we decided to focus solely on the ROI assessments.

In the preregistration, we stated that we would investigate spatial aspects of reliability via x-, y-, and z-coordinates. However, for the sake of brevity we decided to instead employ Dice coefficients (i.e. a measure of spatial overlap), as we deemed them a more succinct and comprehensive representation of our data, also considering the complexity of the manuscript.

In the preregistration, we stated that we would assess reliability only in the ipsilateral dorsal horn of spinal cord segment C6. However, after observing robust activation extending to areas outside the gray matter, we chose to also investigate a larger region encompassing the draining vein territory.

Any other analyses carried out here, but not included in the preregistration, are clearly indicated as post-hoc analyses in the manuscript (see section 2.8).
